# Tuberculose ganglionnaire: aspects épidémiologiques, diagnostiques et thérapeutiques, à propos de 357 cas

**DOI:** 10.11604/pamj.2014.19.157.4916

**Published:** 2014-10-16

**Authors:** Ghizlane Hamzaoui, Lamyae Amro, Hafsa Sajiai, Hind Serhane, Nezha Moumen, Abdellah Ennezari, Abdelhaq Alaoui Yazidi

**Affiliations:** 1 Service de Pneumologie, CHU Mohammed VI, Laboratoire PCIM, FMPM, Université Cadi Ayyad, Marrakech, Maroc; 2 Centre de Diagnostic et de Traitement des Maladies Respiratoires, Marrakech, Maroc

**Keywords:** Tuberculose, ganglion, immunodépression, Tuberculosis, lymph node, immunosuppression

## Abstract

La tuberculose ganglionnaire (TG) est la localisation extrapulmonaire la plus fréquente au Maroc. Elle pose encore un problème diagnostique et thérapeutique. Le but du travail est d’ étudier le profil épidémiologique, diagnostique et thérapeutique de la tuberculose ganglionnaire. Il s'agit d'une étude rétrospective portant sur les nouveaux cas de TG suivis au centre spécialisé de tuberculose de Marrakech, entre Janvier 2011 et Décembre 2012. Trois cents cinquante sept cas de TG ont été inclus sur l'ensemble de 1717 cas de tuberculose toute forme confondue, soit une incidence de 20,8%. La moyenne d’âge était de 29,1 ans avec un sex ratio de 0,6 (62,5% de femmes). Le diabète, le contage tuberculeux et l'infection VIH ont été retrouvés respectivement dans 9%, 14,6% et 3,6% des cas. Les adénopathies étaient cervicales dans 95%, médiastinales dans 5,1%, abdominales dans 3,7%, axillaires dans 2,8% et inguinales dans 0,3% des cas. La radiographie du thorax (faite dans 96,4% des cas) a été anormale dans 8,1%. Le diagnostic a été confirmé dans 97,2% des cas. Le régime thérapeutique était 2 RHZE/4RH dans 88% des cas. Dans les cas suivis, l’évolution a été marquée par la disparition des adénopathies dans 95,2% et par l'augmentation du volume ganglionnaire dans 4,8%. 1,4% des cas ont été perdus de vue. La rechute de TG a été notée dans 3,1%. La TG reste fréquente et occupe la 2^ème^ place après l'atteinte pulmonaire et pose un problème diagnostique et thérapeutique.

## Introduction

La tuberculose constitue un problème mondial de santé publique [1]. Environ un tiers de la population mondiale est infecté par le bacille tuberculeux et plus de neuf millions de nouveaux cas de tuberculose apparaissent dans le monde chaque année. Au Maroc, 26000 à 27000 nouveaux cas de tuberculose toute forme sont dépistés annuellement. La tuberulose extrapulmonaire représente 46% des cas de tuberculose et elle est dominée par l'atteinte ganglionnaire et pleurale qui représente 70% des formes extrapulmonaires [2]. L'objectif de notre étude est d’étudier le profil épidémiologique, diagnostique et thérapeutique de la tuberculose ganglionnaire.

## Méthodes

Nous avons mené une étude rétrospective portant sur les nouveaux cas de TG suivis au centre spécialisé de tuberculose de Marrakech, entre Janvier 2011 et Décembre 2012.

## Résultats

Parmi 1717 cas de tuberculose, on a noté 357 cas de TG, soit une incidence de 20,8%. La TG représentait 38% des nouveaux cas de tuberculose extrapulmonaire. La moyenne d’âge était de 29,1 ans avec un sex ratio de 0,6 (62,5% de femmes). Le diabète, le contage tuberculeux et l'infection VIH ont été retrouvés respectivement dans 9%, 14,6% et 3,6% des cas. L'IDR à la tuberculine faite chez 42,3% des patients, a été positive dans 66,9% des cas avec une moyenne de l'induration de 10,7 mm (extrêmes de 10-23 mm). Les adénopathies (fistulisées dans 2%) étaient cervicales dans 95%, médiastinales dans 5,1%, abdominales dans 3,7%, axillaires dans 2,8% et inguinales dans 0,3% des cas (Tableau 1 et Tableau 2). La radiographie du thorax (faite dans 96,4% des cas) a été anormale dans 8,1% avec présence d'adénopathies thoraciques dans 50%, d'atteinte parenchymateuse dans 28,6% et de pleurésie dans 7,1%.

**Tableau 1:** Caractéristiques épidémiologiques des patients présentant une tuberculose ganglionnaire

Données épidémiologiques		Nombre (pourcentage)
Genre	Hommes	134 (37,5%)
	Femmes	223 (62,5%)
Moyenne d'age		29,1 (2mois -88 ans)
Origine Géographie	Rurale	7 (2%)
	Urbaine	350 (98%)
Profession	Sans profession	151 (42%)
	Etudiants	138 (39%)
	Ouvriers	41 (11%)
	Fonctionnaires	27 (8%)
Tabagisme		33 (9%)
Contage tuberculeux		52 (14,6%)
Comorbidités associées	Diabète	32 (9%)
	VIH	13 (3,6%)
	Insuffisance rénale	7 (2%)

**Tableau 2:** Siège des adénopathies tuberculeuses

Topographie des ganglions	Nombre (pourcentage)
Cervicale isolée	317 (88,8%)
Axillaire isolée	6(1,7%)
Médiastinale isolée	6(1,7%)
abdominale isolée	4(1,1%)
Cervicale et axillaire	3(0,8%)
Cervicale et inguinale	1(0,3%)
Cervicale et médiastinale	11(3,1%)
Cervicale et abdominale	7(1,9%)
Axillaire et abdominale	1(0,3%)
Mediastinale et abdominale	1(0,3%)

**Figure 1:**
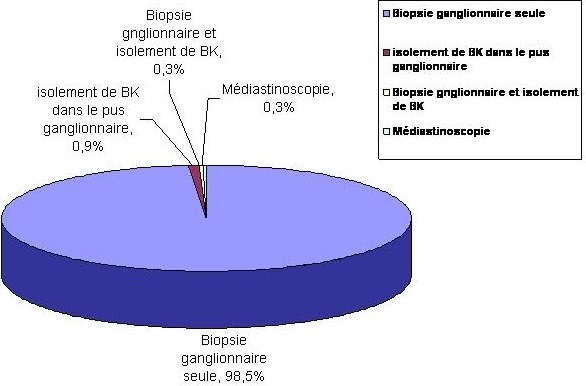
Moyens de confirmation de la tuberculose ganglionnaire

Les associations entre adénopathies thoraciques et autres atteintes notamment l'atteinte pleurale, parenchymateuse et l'atélectasie ont été retrouvées, respectivement, dans 7,1%, 3,6% et 3,6%. La recherche de BK dans les expectorations a été faite dans 17% des cas, elle a été positive dans 6,5% des cas. Le diagnostic a été confirmé dans 97,2% des cas par: la biopsie ganglionnaire seule dans 98,5%, l'isolement de BK dans le pus ganglionnaire dans 0,9%, l'association des deux moyens dans 0,3% et la médiastinoscopie dans 0,3% des cas) (Figure 1). Les patients ont été mis sous traitement antibacillaire selon le programme national de lutte anti-tuberculeuse [3]. Le régime était celui des nouveaux cas de tuberculose (2 RHZE/4RH) dans 88% des cas. Dans les cas suivis, l’évolution a été marquée par la disparition des adénopathies dans 95,2% et par l'augmentation du volume ganglionnaire dans 4,8% ce qui a nécessité la prolongation du traitement antibacillaire de TG. On notait 1,4% de perdus de vue. La rechute de TG a été notée dans 3,1%.

## Discussion

La tuberculose ganglionnaire constitue avec l'atteinte pleurale, une des formes les plus fréquentes de tuberculose extrapulmoanire [3]. La prévalence de la TG est plus élevée dans les pays en développement par rapport aux pays développés. Elle est de 69,5% dans des études réalisées en Ethiopie et en Tanzanie [4]. Nous avons constaté que la majorité des patients présentant une tuberculose ganglionnaire était jeune avec une légère prédominance féminine ce qui concorde avec les résultats d'autres séries (Tableau 3) [5, 6]. Les aires ganglionnaires les plus fréquemment affectées sont les aires cervicales avec une fréquence variant de 47% à 75% selon les études [3, 4]. La tuberculose extrapulmonaire fait suite à une dissemination bacillaire à point de départ pulmonaire [7]. Une étude a montré que 15% des patients se présentaient d'emblée avec une tuberculose pulmonaire concomitante [3], ce pourcentage est augmenté dans notre série (28,6%) d'où l'importance de toujours effectuer une radiographie de thorax (voire au moindre doute un scanner) et des cultures d'expectorations induites ou spontanées lors du bilan d'adénites présumées tuberculeuses.

**Tableau 3:** Comparaison des caractéristiques épidémiologiques avec d'autres séries africaines

Caractéristiques épidémiologiques		Notre série	Bouayad et al [5]	Ait Bachir et al [6]	Muluye et al [4]
Nombre de cas		357	240	101	2392
La moyenne d'age		29,1 ans	26 ans	37 ans	
Sexe		62,5% Femmes	60,41% femmes	64% Femmes	54,1% Femmes
Contage tuberculeux		14,6%	8,75%	11%	
Diabète		9%	1,25%	2%	
Localisation des adénopathies	Cervicales	94,9%	88,7%	60%	70,6%
	Axillaires	2%	5,4%	8%	19,4%
	Inguinales	0,3%	1,2%	2%	6,3%

Le diagnostic différentiel de l'adénite périphérique isolée est large et comprend les syndromes lymphoprolifératifs, l'infection par des mycobactéries non tuberculeuses, la maladie des griffes de chat, les infections fongiques, la toxoplasmose ou les adénites bactériennes [3]. Le diagnostic repose essentiellement sur la cytologie ou l'histologie, rarement sur l’étude bactériologique ceci est dû au caractère paucibacillaire de la tuberculose ganglionnaire, lié à la mauvaise oxygénation des ganglions et à l'importance des mécanismes de défense à médiation cellulaire à ce niveau. La microbiologie standard reste nécessaire puisqu'elle permet l'isolement et l’étude de la sensibilité du Mycobacterium [8]. Le traitement des adénites tuberculeuses, étayé par une étude de la British Thoracic Society Research Committee, repose sur une quadrithérapie antituberculeuse classique d'isoniazide, rifampicine, éthambutol et pyrazinamide (HREZ) deux mois, puis isoniazide et rifampicine (HR) quatre mois, avec un taux de rechute de l'ordre de 3% ce qui concorde avec nos résultas [3]. L'augmentation paradoxale du volume de l'adénite ou l'apparition de nouveaux ganglions, qu'ils soient proches ou à distance du site primaire, sont des complications relativement fréquentes des atteintes ganglionnaires tuberculeuses (10–22%), dans notre série elle a été plus basse (4,8%) [3].

## Conclusion

Notre étude a montré l'incidence élevée de la tuberculose ganglionnaire. Le diagnostic repose sur la cytohistologie, la microbiologie standard reste l'examen clé permettant de déterminer l'agent causale de la tuberculose ganglionnaire et de tester sa sensibilité au traitement antibacillaire. Les auteurs ne déclarent aucun conflit d'intérêt.
